# Effect of Added Salt on the RAFT Polymerization of 2-Hydroxyethyl Methacrylate in Aqueous Media

**DOI:** 10.1021/acs.macromol.4c01078

**Published:** 2024-07-12

**Authors:** Csilla György, Jacob S. Wagstaff, Saul J. Hunter, Esther U. Etim, Steven P. Armes

**Affiliations:** † Dainton Building, Department of Chemistry, Brook Hill, University of Sheffield, Sheffield, South Yorkshire S3 7HF, U.K.; ‡ Joseph Banks Laboratories, School of Chemistry, University of Lincoln, Lincolnshire LN6 7TS, U.K.

## Abstract

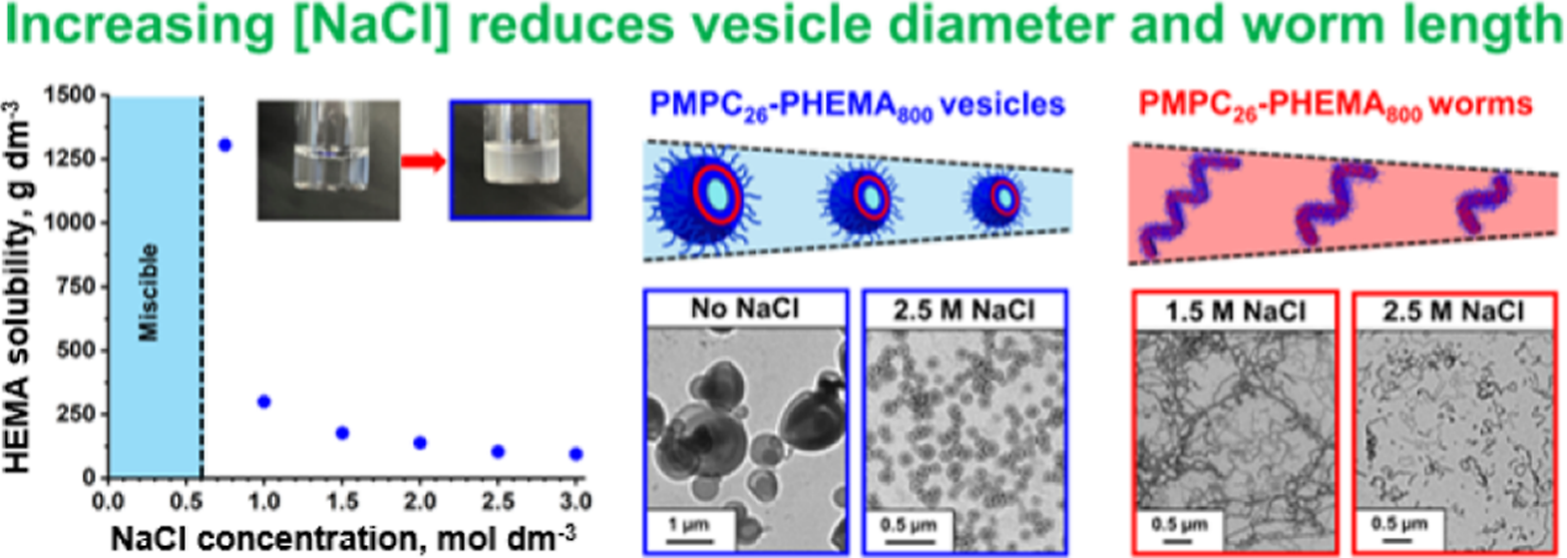

We report the effect of added salt on the reversible addition–fragmentation chain transfer (RAFT) polymerization of 2-hydroxyethyl methacrylate (HEMA) in aqueous media. More specifically, poly(2-(methacryloyloxy)ethyl phosphorylcholine) (PMPC_26_) was employed as a salt-tolerant water-soluble block for chain extension with HEMA targeting PHEMA DPs from 100 to 800 in the presence of NaCl. Increasing the salt concentration significantly reduces the aqueous solubility of both the HEMA monomer and the growing PHEMA chains. HEMA conversions of more than 99% could be achieved within 6 h at 70 °C regardless of the NaCl concentration when targeting PMPC_26_-PHEMA_800_ vesicles at 20% w/w solids. Significantly faster rates of polymerization were observed at higher salt concentration owing to the earlier onset of micellar nucleation. Transmission electron microscopy (TEM) was used to construct a pseudo-phase diagram for this polymerization-induced self-assembly (PISA) formulation. High-quality images required cross-linking of the PHEMA chains with glutaraldehyde prior to salt removal via dialysis. Block copolymer spheres, worms, or vesicles can be accessed at any salt concentration up to 2.5 M NaCl. However, only kinetically trapped spheres could be obtained in the presence of 3 M NaCl because the relatively low HEMA monomer solubility under such conditions leads to an aqueous emulsion polymerization rather than an aqueous dispersion polymerization. In this case, dynamic light scattering studies indicated a gradual increase in *z*-average diameter from 26 to 86 nm when adjusting the target PHEMA degree of polymerization from 200 to 800. When targeting PMPC_26_-PHEMA_800_ vesicles, increasing the salt content up to 2.5 M NaCl leads to a systematic reduction in the *z*-average diameter from 953 to 92 nm. Similarly, TEM analysis and dispersion viscosity measurements indicated a gradual reduction in worm contour length with increasing salt concentration for PMPC_26_-PHEMA_600_ worms. This new PISA formulation clearly illustrates the importance of added salt on aqueous monomer solubility and how this affects (i) the kinetics of polymerization, (ii) the morphology of the corresponding diblock copolymer nano-objects, and (iii) the mode of polymerization in aqueous media.

## Introduction

It is well documented that controlled radical polymerization techniques such as reversible addition–fragmentation chain transfer (RAFT) polymerization enable the convenient synthesis of a remarkably broad range of functional vinyl polymers.^1−7^ When combined with polymerization-induced self-assembly (PISA), RAFT polymerization offers the opportunity to develop rational syntheses of many types of diblock copolymer nano-objects in various solvents.^8−18^ In essence, PISA simply involves growing a second insoluble block from a soluble precursor block in a suitable selective solvent to afford sterically stabilized nano-objects.^19,20^ Aqueous syntheses are particularly prevalent in the PISA literature, no doubt because water is cheap, non-toxic, and potentially amenable to industrial scale-up. Moreover, such formulations are well suited to various bioapplications.^21−28^

Depending on whether the vinyl monomer is water-miscible or water-immiscible, heterogeneous PISA formulations can be classified as either RAFT aqueous dispersion polymerization or RAFT aqueous emulsion polymerization, respectively.^20,29−49^ In the former case, various copolymer morphologies (e.g., spheres, worms, or vesicles) can be readily accessed.^20,50−54^ In contrast, aqueous emulsion polymerization formulations often lead to kinetically trapped spheres^34−37,55−59^ regardless of the target diblock copolymer composition, although there are various well-known exceptions.^8,31,32,38,60−62^ Recently, we postulated that the aqueous monomer solubility should be an important parameter in this context. Indeed, we found that water-immiscible vinyl monomers with moderate aqueous solubility such as 2-methoxyethyl methacrylate, glycidyl methacrylate, or hydroxybutyl methacrylate provide access to spheres, worms, or vesicles.^63−68^ On the other hand, monomers such as styrene, *n*-butyl acrylate, benzyl methacrylate, or 2,2,2-trifluoroethyl methacrylate exhibit lower aqueous solubility (≤1 g dm^–3^) and usually form kinetically trapped spheres.^34−37,55−59^

**Figure 1:**
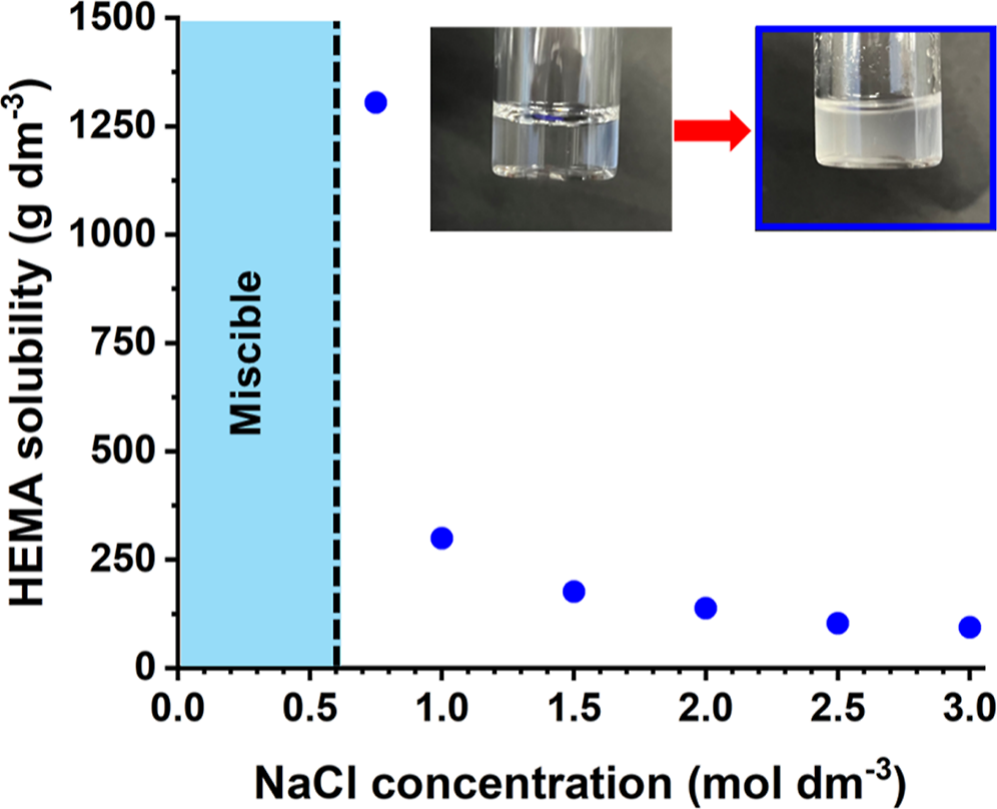
Aqueous solubility of HEMA monomer as a function of added salt at 70 °C. HEMA is water-miscible in all proportions in the presence of up to 0.6 M NaCl. Inset: digital images recorded for the aqueous homogeneous mixture comprising HEMA and 1.5 M NaCl formed at [HEMA] = 171 g dm^−3^ (left) and for the aqueous emulsion produced at [HEMA] = 176 g dm^–3^ (right).

Given the above literature precedent, it would be interesting to identify an aqueous PISA formulation in which the solubility of the vinyl monomer could be systematically varied. In the present study, we report such a formulation: the addition of salt (NaCl) enables the aqueous solubility of 2-hydroxyethyl methacrylate (HEMA) to be tuned over a wide range (see Figure 1). One key aspect of this new aqueous PISA formulation is the choice of the steric stabilizer precursor, which must be highly tolerant of added salt. In view of this constraint, we chose to use poly(2-(methacryloyloxy)ethyl phosphorylcholine) (PMPC), which has been employed for various RAFT aqueous dispersion polymerization syntheses^30,69,70^ and is known to remain water-soluble even in the presence of 5 M NaCl.^71^

In the absence of any added salt, HEMA monomer is fully miscible with water in all proportions. Thus, given that poly(2-hydroxyethyl methacrylate) (PHEMA) becomes water-insoluble above a relatively low degree of polymerization (DP),^72^ the RAFT polymerization of HEMA in aqueous media should produce diblock copolymer nanoparticles. Herein, we examine the RAFT synthesis of PMPC_26_-PHEMA_*x*_ nanoparticles in neutral aqueous media (pH 6.3) in the presence of up to 3 M NaCl. This new aqueous PISA formulation offers an opportunity to systematically study the effect of added salt on the HEMA polymerization kinetics and the resulting copolymer morphology.

## Experimental Section

### Materials

2-(Methacryloyloxy)ethyl phosphorylcholine (MPC) was obtained from NOF Corporation (Japan) and was used as received. 2-Cyano-2-propyl benzodithioate (CPDB; >97%), 4,4′-azobis(4-cyanopentanoic acid) (ACVA; 99%), glutaraldehyde (GA; supplied as a 50% w/w aqueous solution), and pyridine were purchased from Merck (UK), while 2,2′-azobis(isobutyronitrile) (AIBN) was purchased from Molekula (UK). 2-Hydroxyethyl methacrylate (HEMA; ≥99.5% triply distilled grade) monomer was kindly provided by GEO Specialty Chemicals (Hythe, UK) and was used as received. Chloroform, methanol, and ethanol were obtained from VWR Chemicals (UK). NaCl (99.5%) was purchased from Fisher Scientific (UK). Deuterated dimethyl sulfoxide (DMSO-*d*_6_; 99.9%) and deuterated methanol (CD_3_OD; 99.8%) were purchased from Cambridge Isotope Laboratories (UK).

### Synthesis of Poly(2-(Methacryloyloxy)ethyl Phosphorylcholine) (PMPC_26_) Precursor via RAFT Solution Polymerization of MPC in Ethanol

This synthesis protocol was previously reported by Beattie et al.^37^ MPC monomer (35.0 g, 0.11 mol), CPDB (937.0 mg, 4.23 mmol), and AIBN initiator (139.0 mg, 0.85 mmol, CPDB/AIBN molar ratio = 5.0) were dissolved in ethanol (54.11 g) to afford a 40% w/w solution in a sealed round-bottom flask containing a magnetic stir bar. This flask was immersed in an ice bath, and the reaction mixture was deoxygenated with a stream of N_2_ gas for 30 min. The flask was heated to 70 °C with magnetic stirring for 140 min, and then the MPC polymerization was quenched by exposing the reaction mixture to air while cooling the flask to 20 °C. A final MPC conversion of 82% was determined by comparing the integrated vinyl proton signal at 5.65–6.20 ppm with the oxymethylene signals assigned to the polymerized MPC units at 4.0–4.4 ppm using ^1^H NMR spectroscopy. The crude PMPC was precipitated twice into a 10-fold excess of a 17:1 v/v acetone/methanol mixture. Then the purified precursor was redissolved in deionized water and freeze-dried overnight to produce a pink solid. The mean DP was determined to be 26 via ^1^H NMR spectroscopy by comparing the five aromatic phenyl protons assigned to the dithiobenzoate end group at 7.45–8.00 ppm with the two azamethylene protons assigned to the polymerized MPC units at 3.75 ppm. Gel permeation chromatography (GPC) studies indicated an *M*_n_ of 3.5 kg mol^–1^ and an *M*_w_/*M*_n_ of 1.19 when using an aqueous eluent and an *M*_n_ of 3.6 kg mol^–1^ and an *M*_w_/*M*_n_ of 1.32 when using a 3:1 chloroform/methanol eluent (see below for further GPC details).

### Effect of Added NaCl on the Aqueous Solubility of HEMA Monomer at 70 °C

Deionized water (2.0 g) was added to a pre-weighed vial equipped with a magnetic stir bar. This vial was placed in an oil bath set at 70 °C and allowed to equilibrate for 20 min. HEMA was added to a second pre-weighed vial and then added dropwise to the first vial at 70 °C. After the addition of each drop of HEMA, the aqueous HEMA mixture was stirred at 70 °C for 1 min. Visual inspection was used to judge the point at which the HEMA monomer droplets were no longer fully dissolved. At this point, the vial containing the remaining HEMA monomer was reweighed to calculate the total mass of added HEMA and hence determine its aqueous solubility at 70 °C. This experiment was repeated with the deionized water being replaced with a series of aqueous salt solutions (up to 3 M NaCl).

### In Situ Kinetic Study of the Synthesis of PMPC_26_-PHEMA_800_ Nanoparticles via RAFT Aqueous Polymerization of HEMA in the Presence of 0–3 M NaCl

PMPC_26_ precursor (30.0 mg, 3.75 μmol), ACVA (0.21 mg, 0.75 μmol), and HEMA (0.39 g, 3.00 mmol) were mixed in turn with a series of aqueous solutions (1.68 g) containing 0, 1.5, 2.5, or 3 M NaCl to target PMPC_26_-PHEMA_800_ nanoparticles. Each reaction mixture was purged with N_2_ in a sealed reaction vessel, and ∼0.40 mL was placed in an NMR tube equipped with a J-Young’s tap under a N_2_ atmosphere along with a sealed inner capillary tube containing pyridine dissolved in DMSO-*d*_6_, which served as an external standard. A reference NMR spectrum was recorded at 20 °C. The NMR tube was then heated to 70 °C within the spectrometer to initiate the HEMA polymerization. Spectra were recorded at ∼5 min intervals. The instantaneous HEMA conversion was determined by monitoring the progressive reduction in the HEMA vinyl signals at 4.80–5.90 ppm relative to that of the five aromatic pyridine proton signals at 7.25–8.68 ppm.

### RAFT Aqueous Polymerization of HEMA Targeting PMPC_26_-PHEMA_100_ Nanoparticles

A stock solution comprising HEMA and ACVA (2.0 g; HEMA/ACVA molar ratio = 500) was prepared in a glass vial. An aliquot of this stock solution (0.250 g, containing 1.88 mmol HEMA and 3.75 μmol ACVA; target DP = 100) and PMPC_26_ precursor (0.150 g, 18.75 μmol, PMPC_26_/ACVA molar ratio = 5.0) was weighed into a glass vial equipped with a magnetic stir bar. An aqueous solution (1.58 g) containing 0–3 M NaCl was added to target a final copolymer concentration of 20% w/w solids, and the reaction mixture was degassed using N_2_ gas for 30 min. The sealed reaction vessel was then heated to 70 °C for 6 h. When targeting higher DPs, the total mass was always maintained at approximately 2.0 g by adjusting the relevant reactant masses as required. In each case, relatively high monomer conversions were confirmed by ^1^H NMR analysis, as indicated by the disappearance of the HEMA vinyl signals at 5.65–6.20 ppm.

### Crosslinking of PMPC_26_-PHEMA_*x*_ Nanoparticles for Transmission Electron Microscopy Analysis

The crosslinking protocol used herein was recently reported by Deane et al.^53^ Glutaraldehyde (GA, 20 μL of a 50% aqueous solution, 0.20 mmol) was added to a 5.0% w/w aqueous copolymer dispersion (2.0 mL; GA/HEMA molar ratio = 0.30) that had been diluted to maintain its original NaCl concentration. After stirring for 24 h at 20 °C, a second aliquot of GA (0.20 mmol, 20 μL) was added, and crosslinking was continued for a further 2 h. The resulting aqueous dispersion of core-crosslinked nanoparticles was dialyzed against the corresponding aqueous solution (0–3 M NaCl) for at least 48 h prior to transmission electron microscopy (TEM) grid preparation. Furthermore, dynamic light scattering (DLS) analysis indicated no significant change in copolymer morphology before and after crosslinking (see Table S1).

### ^1^H NMR Spectroscopy

^1^H NMR spectra were recorded for the aqueous diblock copolymer dispersions diluted in CD_3_OD using a 400 MHz Bruker Avance spectrometer. Typically, 64 scans were averaged per spectrum. During the in situ ^1^H NMR kinetic experiment, spectra were acquired in eight transients using a 30° excitation pulse and a delay time of 5 s over a spectral window of 16 kHz with 64 k data points.

### Gel Permeation Chromatography

If required, the as-synthesized aqueous copolymer dispersions were dialyzed for 48 h to remove salt prior to GPC analysis. The resulting salt-free aqueous dispersions were then freeze-dried overnight to remove water. GPC analysis was conducted at 35 °C using a 3:1 v/v chloroform/methanol eluent containing 2 mM LiBr at a flow rate of 1.0 mL min^–1^. The instrument setup comprised an Agilent 1260 GPC system, two Agilent PL gel 5 mm Mixed-C columns connected in series with a guard column, and a refractive index detector. Calibration was achieved using a series of ten near-monodisperse poly(methyl methacrylate) (PMMA) standards with *M*_p_ values ranging from 2380 to 988 000 g mol^–1^.

Aqueous GPC analysis of the PMPC_26_ precursor was conducted at 30 °C using an aqueous eluent containing 0.10 M NaNO_3_, 0.02 M TEA, 0.05 M NaHCO_3_, and 0.005 M NaN_3_ (pH 8) at a flow rate of 1.0 mL min^–1^. The instrument setup comprised an Agilent 1260 GPC system; three PL Aquagel Mixed-H, OH-30, and OH-40 columns connected in series with a guard column; and a refractive index detector. Calibration was achieved using a series of ten near-monodisperse poly(ethylene glycol) (PEG) standards with *M*_p_ values ranging from 240 to 912 800 g mol^–1^.

### Dynamic Light Scattering

DLS studies were performed using a Zetasizer Nano ZS instrument (Malvern Instruments, UK) at a fixed scattering angle of 173°. Copolymer dispersions were diluted to 0.10% w/w solids using aqueous solutions containing 0–3 M NaCl prior to analysis at 20 °C. The *z*-average diameter and polydispersity of the nanoparticles were calculated by cumulant analysis of the experimental correlation function using Dispersion Technology Software version 6.20. Data were averaged over ten runs each of 30 s duration. Increasing the salt concentration leads to a significant increase in the aqueous solution viscosity. Hence, literature data for the viscosity of 0.5–3 M NaCl aqueous solutions^73^ were used when calculating *z*-average diameters using the Stokes–Einstein equation.

### Transmission Electron Microscopy

Aqueous dispersions of glutaraldehyde-cross-linked nanoparticles were diluted to 0.10% w/w using a series of 0.5–3 M aqueous NaCl solutions after dialysis. In the absence of added salt, no core-crosslinking was required for TEM analysis. Copper–palladium TEM grids were surface-coated with a thin carbon film before being plasma glow-discharged for 30 s to produce a hydrophilic surface. An 8 μL droplet of a dilute aqueous dispersion of PMPC_26_-PHEMA_*y*_ nanoparticles was deposited onto the surface of each TEM grid for 1 min before blotting with filter paper to remove excess liquid. An 8 μL droplet of a 0.75% w/v aqueous uranyl formate solution was then applied as a negative stain for 25 s prior to careful blotting and drying using a vacuum hose. Imaging was performed at 80 kV using a FEI Tecnai G2 spirit instrument equipped with a Gatan 1k CCD camera.

### Dispersion Viscosity Measurements

An Anton Paar MCR 502 rheometer equipped with a 50 mm 2° stainless steel cone was used with a sample gap of 207 μm. Rotational rheometry was used at a fixed shear rate of 10 s^–1^ to determine the viscosity for selected dispersions comprising diblock copolymer worms.

## Results and Discussion

### Effect of Added NaCl on the Aqueous Solubility of HEMA Monomer at 70 °C

HEMA monomer is fully miscible with water in all proportions. However, its aqueous solubility strongly depends on the presence of salt. For example, the digital photograph shown in Figure 1 (blue frame) clearly illustrates the transition from fully soluble HEMA in 0–0.6 M NaCl to (partially) immiscible HEMA in the presence of 1.5 M NaCl. This change in physical appearance is used as the “end point” for a series of gravimetric titrations to determine the aqueous solubility of HEMA in various salt solutions at 70 °C. For example, HEMA solubility is reduced from 1305 g dm^–3^ in 0.75 M NaCl to just 93 g dm^–3^ in 3 M NaCl (see Figure 1). In principle, this should be sufficient for the mode of polymerization to switch from an aqueous dispersion polymerization to an aqueous emulsion polymerization. Moreover, added salt was also expected to lower the critical DP at which PHEMA chains become water-insoluble. Combining the salt-tunable aqueous solubility of HEMA with a suitable salt-tolerant precursor such as PMPC should facilitate a systematic study of the effect of added salt on the polymerization kinetics and copolymer morphology.

### Synthesis and Characterization of the PMPC_26_ Precursor and PMPC_26_-PHEMA_*x*_ Diblock Copolymers

**Scheme 1:**
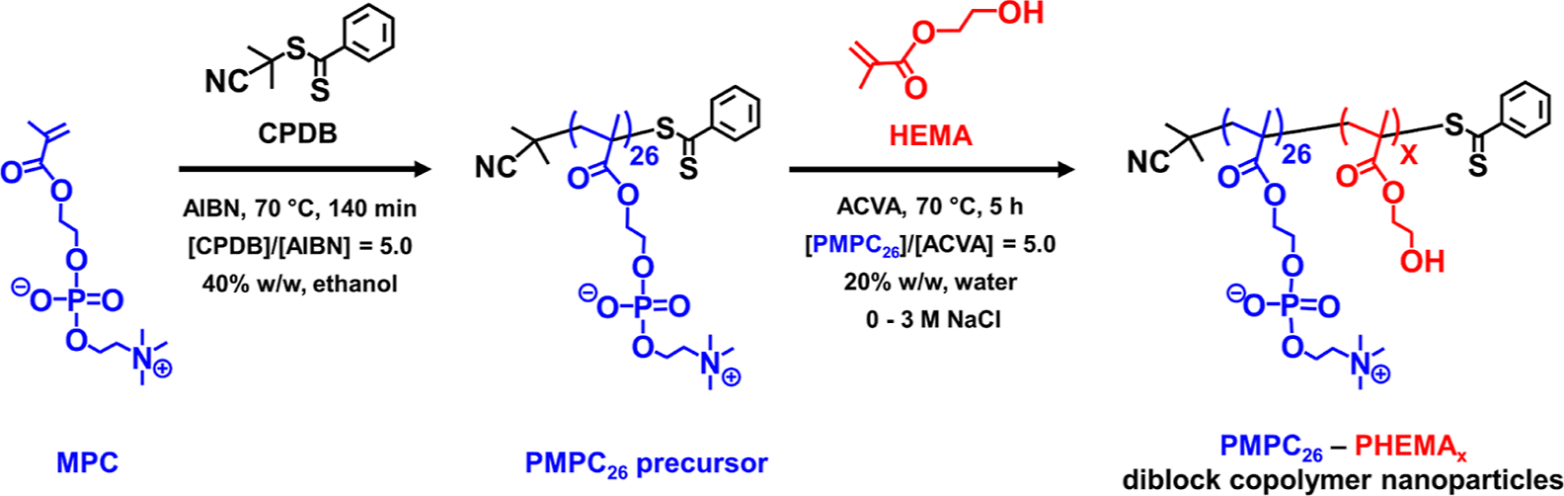
Synthesis of Poly(2-(methacryloyloxy)ethyl phosphorylcholine) (PMPC_26_) via RAFT Solution Polymerization of MPC in Ethanol at 40% w/w Solids Using 2-Cyano-2-propyl Dithiobenzoate (CPDB) RAFT Agent and 2,2′-Azobisisobutyronitrile (AIBN) Initiator at 70 °C; This Precursor Was Chain-Extended by RAFT Aqueous Polymerization of 2-Hydroxyethyl Methacrylate (HEMA) Targeting 20% w/w Solids at 70 °C in the Presence of 0–3 M NaCl

**Figure 2:**
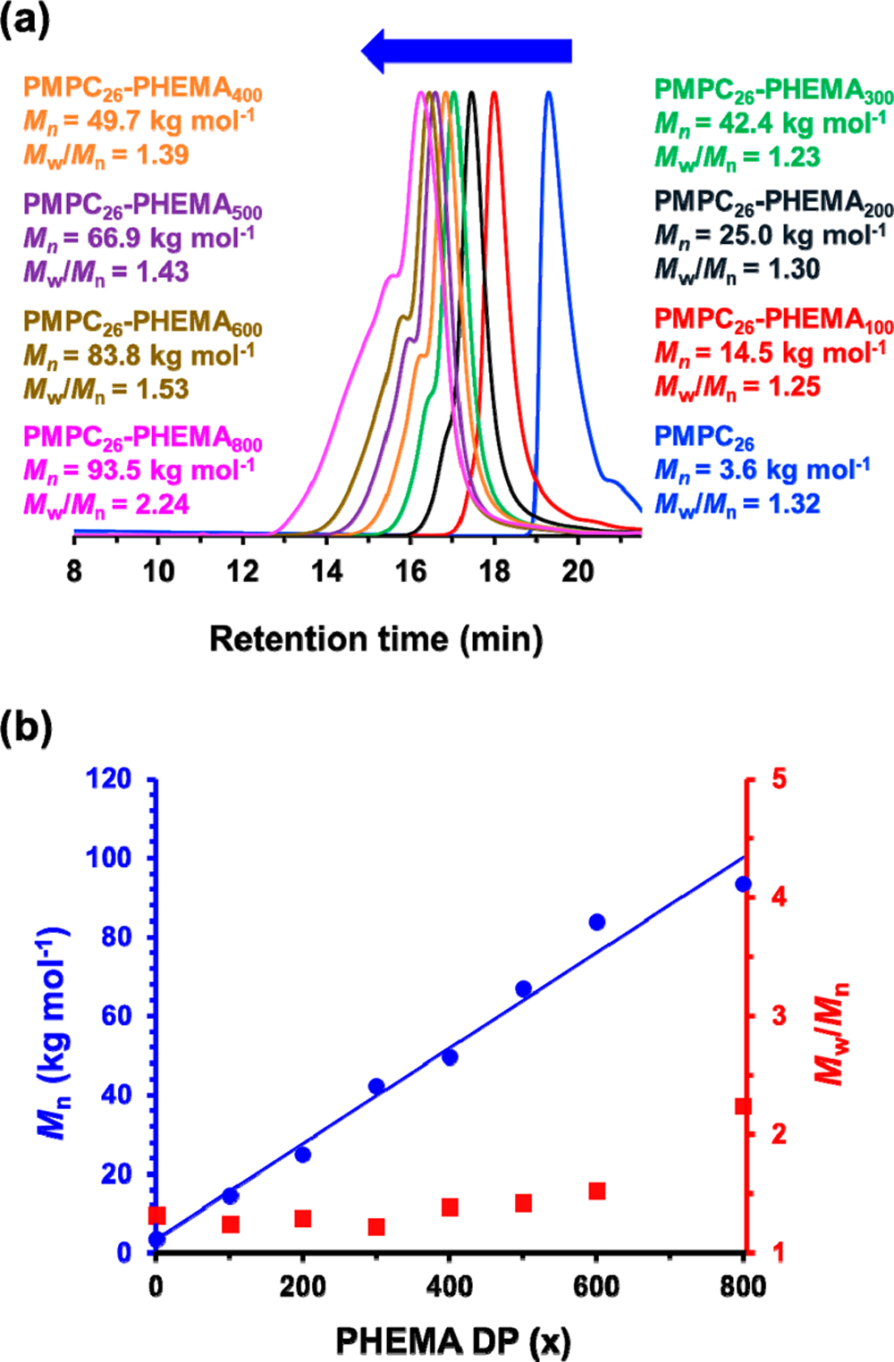
(a) Gel permeation chromatograms (vs a series of near-monodisperse poly(methyl methacrylate) calibration standards using a refractive index detector) obtained for the PMPC_26_ precursor (prepared in ethanol at 40% w/w solids at 70 °C) and a series of PMPC_26_-PHEMA_100–800_ diblock copolymers prepared by RAFT aqueous dispersion polymerization of HEMA at 70 °C targeting 20% w/w solids in the presence of 1 M NaCl. (b) Linear relationship between *M*_n_ (blue circles) and PHEMA DP for the same PMPC_26_-PHEMA_100–800_ series. The corresponding *M*_w_/*M*_n_ (red squares) data are also shown.

One aim of this study is to examine the effect of added salt on the nanoparticle morphology. According to the PISA literature,^51,74^ accessing higher order morphologies requires the use of a sufficiently short steric stabilizer. Recently, we reported that a PMPC_26_ precursor was required to access either worms or vesicles via the RAFT aqueous dispersion polymerization of 2-hydroxypropyl methacrylate (HPMA).^69^ Given that HEMA and HPMA have similar chemical structures, we elected to use the same PMPC_26_ precursor in the present study. A suitable dithiobenzoate-based RAFT agent, 2-cyano-2-propyl dithiobenzoate (CPDB), and 2,2′-azobis(isobutyronitrile) (AIBN) initiator were employed for the RAFT solution polymerization of MPC in ethanol at 70 °C (see Scheme 1), as previously reported by Beattie et al.^69^ A ^1^H NMR spectrum recorded in CD_3_OD indicated a mean DP of 26 for the resulting PMPC precursor (see Figure S1). Aqueous GPC analysis using a refractive index detector indicated a number-average molecular weight (*M*_n_) of 3.5 kg mol^–1^ with a relatively low dispersity (*M*_w_/*M*_n_ = 1.19), suggesting good RAFT control (see Figure S2). GPC analysis using a 3:1 chloroform/methanol eluent indicated an *M*_n_ of 3.6 kg mol^–1^ with a somewhat higher dispersity (*M*_w_/*M*_n_ = 1.32), see Figure 2. Aqueous GPC analysis is considered more reliable for this precursor, but unfortunately this eluent is not suitable for assessing the chain extension efficiency achieved when preparing PMPC_26_-PHEMA_*x*_ diblock copolymers.

This PMPC_26_ precursor was then chain-extended via RAFT aqueous polymerization of HEMA at 70 °C using 4,4′-azobis(4-cyanovaleric acid) (ACVA) as a water-soluble radical initiator (see Scheme 1). The target PHEMA DP was systematically varied between 100 and 800 while targeting a copolymer concentration of 20% w/w solids and adjusting the NaCl concentration between 0.5 and 3.0 M. More than 99% HEMA conversion was achieved for all syntheses, as confirmed by ^1^H NMR spectroscopy studies. In Figure 2, GPC curves are shown for the PMPC_26_-PHEMA_100–800_ series prepared in the presence of 1 M NaCl. This salt concentration corresponds to a RAFT aqueous dispersion polymerization formulation. Unimodal curves were obtained with relatively narrow molecular weight distributions (*M*_w_/*M*_n_ ≤ 1.30) when targeting PHEMA DPs up to 300 (see Figure 2a). However, targeting higher PHEMA DPs produced significantly broader molecular weight distributions (*M*_w_/*M*_n_ = 1.39–2.24) owing to the appearance of a high molecular weight shoulder (see Figure 2a). Similar observations were also made for the copolymer series prepared in the presence of 2.5 M NaCl, see Figure S3. In principle, dimethacrylate impurities within the HEMA monomer may be responsible for this feature. Indeed, targeting higher PHEMA DPs led to higher copolymer dispersities (see Tables S2–S8). On the other hand, high-purity triply distilled HEMA containing less than 0.10% dimethacrylate was employed for these experiments. Alternative explanations might be chain transfer to polymer or termination by combination, which would inevitably lead to a higher *M*_w_.^75^ However, these latter two mechanisms are relatively unlikely for methacrylic monomers such as HEMA. As expected, a linear evolution in *M*_n_ with increasing PHEMA DP was observed for this PMPC_26_-PHEMA_100–800_ series, see Figure 2.

### In Situ Kinetic Studies of the RAFT Aqueous Polymerization of HEMA in the Presence of 0–3 M NaCl

**Figure 3:**
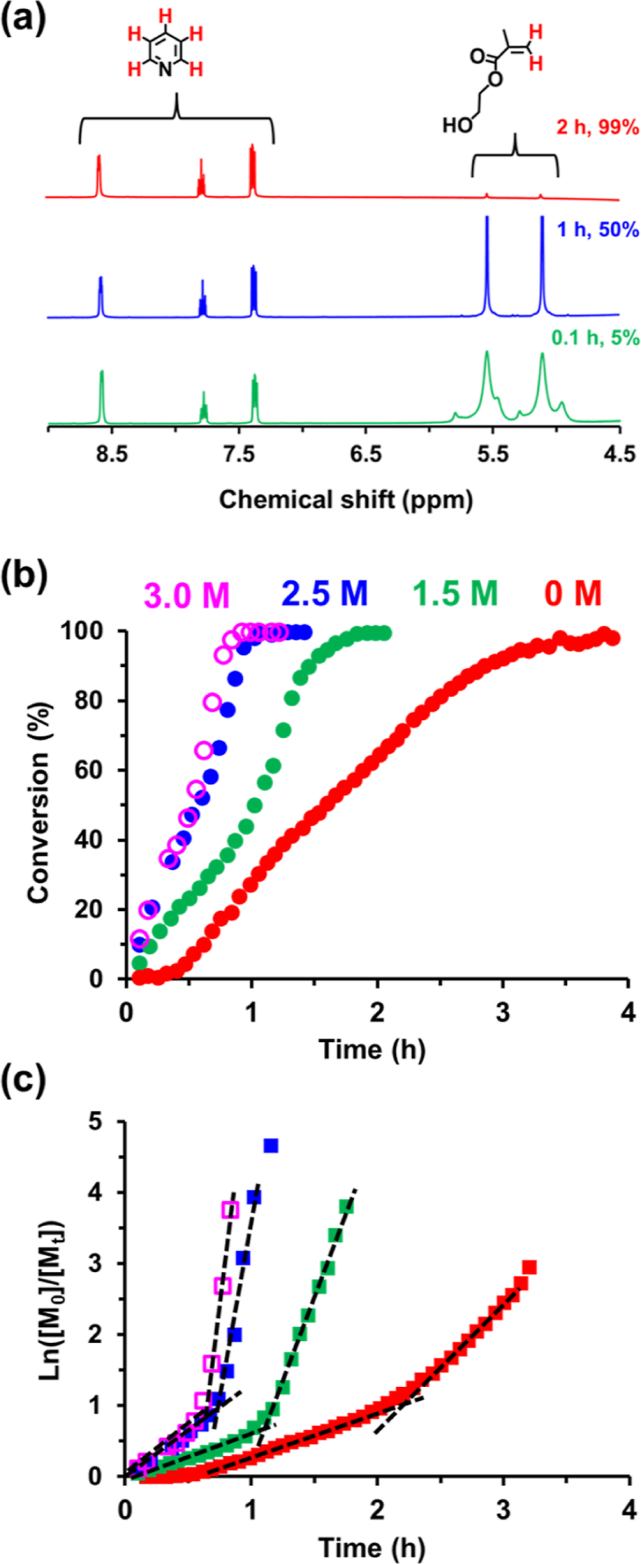
(a) Selected partial ^1^H NMR spectra recorded during the RAFT aqueous polymerization of HEMA at 70 °C when targeting a 20% w/w dispersion of PMPC_26_-PHEMA_800_ vesicles in the presence of 1.5 M NaCl after 0.1 h (green data), 1 h (blue data), and 2 h (red data) using pyridine as an external standard to determine the instantaneous monomer conversion (%). (b) Corresponding conversion vs. time curves obtained during the synthesis of PMPC_26_-PHEMA_800_ vesicles at 70 °C either in the absence of salt (red data) or in the presence of 1.5 M NaCl solution (green data), 2.5 M NaCl solution (blue data), and 3.0 M NaCl solution, respectively. (c) Corresponding semilogarithmic plots for the same aqueous PISA syntheses.

The HEMA polymerization kinetics was monitored in situ by ^1^H NMR spectroscopy during the synthesis of PMPC_26_-PHEMA_800_ nanoparticles at 70 °C when targeting 20% w/w solids in the presence of either no added salt or 1.5 to 3.0 M NaCl, respectively. The monomer conversion was determined over time by monitoring the progressive attenuation of the HEMA vinyl signals relative to the aromatic pyridine signals (see Figure 3). The semilogarithmic plots (see Figure 3b) indicate a significant increase in the rate of polymerization in each case (Figure 3c), which corresponds to the onset of micellar nucleation.^20^ In the absence of any added salt, this rate acceleration occurs at 2.0 h, which corresponds to 67% HEMA conversion or an instantaneous PHEMA DP of approximately 537. For this zero salt formulation, there is a 2.5-fold increase in the rate of polymerization. Increasing the salt concentration up to 1.5 M NaCl leads to micellar nucleation after 1.1 h or 57% HEMA conversion, which corresponds to a PHEMA DP of 452. There is a 6-fold increase in the rate of polymerization at this point. In the presence of 2.5 M NaCl, micellar nucleation occurs after 0.6 h (or 67% HEMA conversion). This corresponds to an instantaneous PHEMA DP of 533 and a 7-fold increase in the rate of polymerization. Finally, micellar nucleation occurs after 0.55 h (or 55% HEMA conversion) for the 3 M NaCl formulation. This corresponds to an instantaneous PHEMA DP of 438 and a 7.5-fold increase in the rate of polymerization. These observations indicate that the onset of micellar nucleation occurs on shorter time scales at higher salt concentrations as this PISA formulation switches from an aqueous dispersion polymerization to an aqueous emulsion polymerization. Moreover, the rate of polymerization both before and after micellar nucleation is always faster at higher salt concentration. In all cases, the initial rate enhancement is eventually followed by a slower rate of polymerization under monomer-starved conditions. Furthermore, conversion vs. time plots reveal that essentially full HEMA conversion (≥99%) could be achieved in all cases (e.g., see Figure 3b), within 4.0 h in the absence of added salt or after 2.0, 1.2, or 1.0 h in the presence of 1.5, 2.5, or 3 M NaCl, respectively.

### Pseudo-phase Diagram Constructed for PMPC_26_-PHEMA_*x*_ Nanoparticles Prepared in the Presence of 0–3 M NaCl

**Figure 4:**
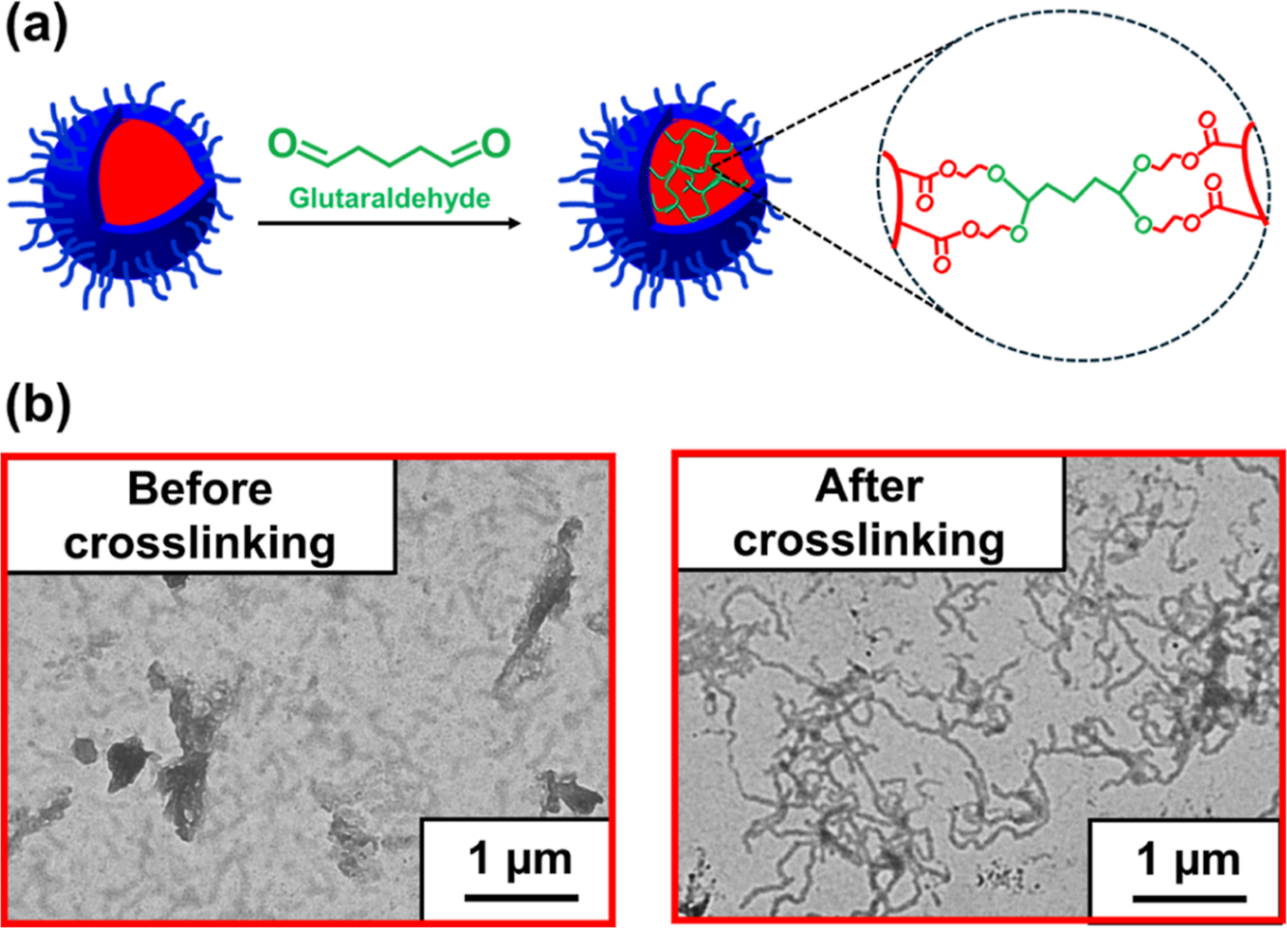
(a) Schematic representation of crosslinking between PHEMA chains when reacting PMPC_26_-PHEMA_*x*_ nanoparticles with glutaraldehyde. (b) Representative TEM images obtained for PMPC_26_-PHEMA_600_ worms prepared at 20% w/w solids in the presence of 1 M NaCl before and after crosslinking with excess glutaraldehyde at 1% w/w copolymer concentration at 25 °C.

There are numerous literature examples of the construction of pseudo-phase diagrams for aqueous PISA formulations on the basis of TEM analysis.^20,51^ Indeed, this systematic approach is essential for the reproducible targeting of pure copolymer morphologies. For example, Baddam et al. reported a partial pseudo-phase diagram when targeting poly[(vinylbenzyl) trimethylammonium chloride]–poly(diacetone acrylamide) (PVBTMAC_27_-PDAAM_248–252_) nanoparticles in the presence of up to 2 M NaCl at 16–18% w/w solids. In this case, adjusting the ionic strength of the aqueous reaction mixture was required to provide access to either spheres or vesicles when using the highly cationic PVBTMAC precursor.^76^ Similarly, we wished to examine the effect of varying the salt concentration on the final copolymer morphology for the PMPC_26_-PHEMA_*x*_ formulation. However, dialysis was required to remove salt prior to TEM analysis; otherwise, salt crystals were formed during TEM grid preparation. To prevent any possible change in copolymer morphology, we decided to crosslink the nanoparticle cores using glutaraldehyde prior to dialysis. This reagent reacts with the primary hydroxyl groups on the PHEMA chains (see Figure 4a). Figure 4b depicts representative TEM images obtained for PMPC_26_-PHEMA_600_ worms before and after core-crosslinking with glutaraldehyde.

**Figure 5:**
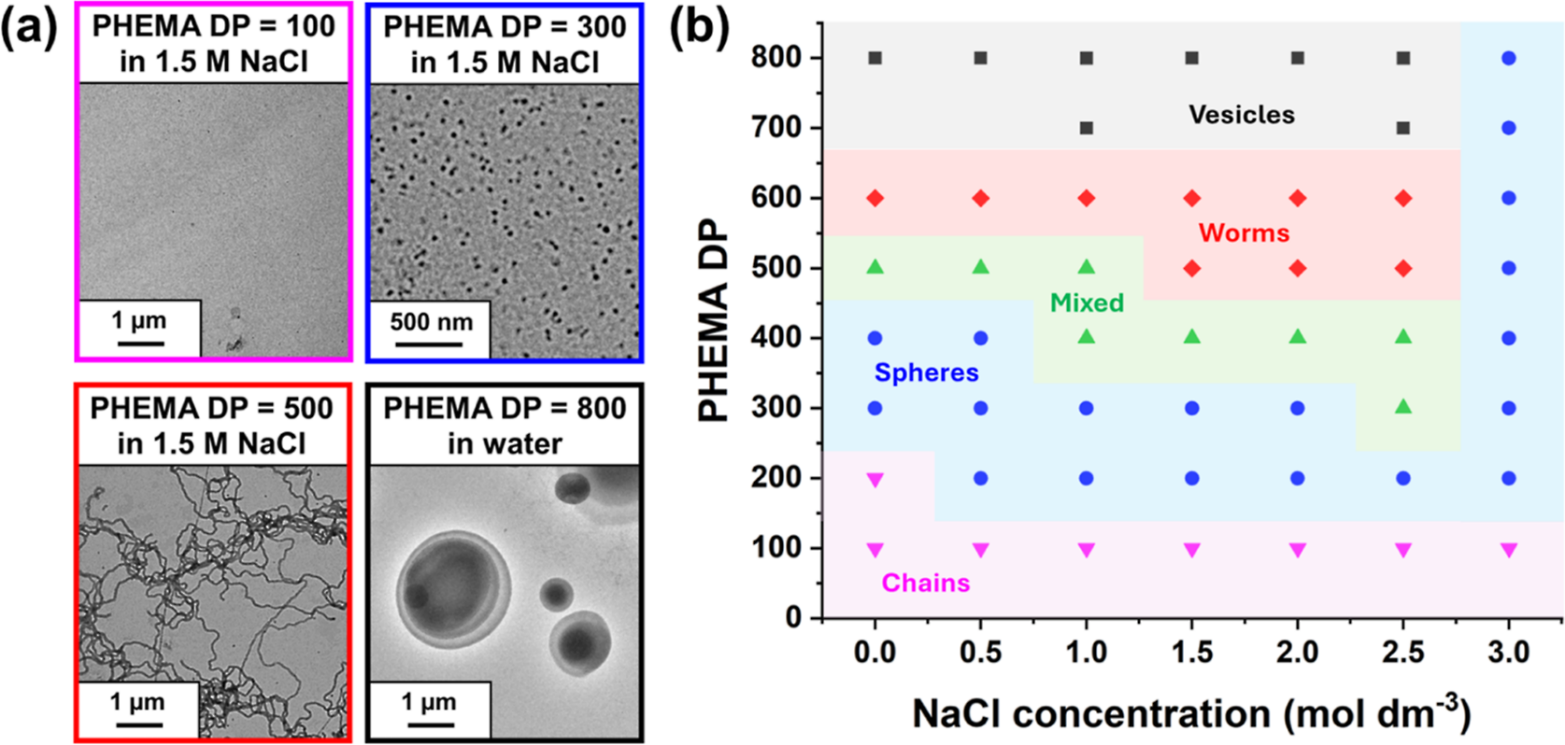
(a) Representative TEM images obtained for molecularly dissolved PMPC_26_-PHEMA_100_ chains (purple frame), PMPC_26_-PHEMA_300_ spheres (blue frame), PMPC_26_-PHEMA_500_ worms (red frame) prepared in the presence of 1.5 M NaCl, and PMPC_26_-PHEMA_800_ vesicles (black frame) prepared in the absence of salt when targeting 20% w/w solids at 70 °C. (b) Pseudo-phase diagram constructed for PMPC_26_-PHEMA_*x*_ nanoparticles prepared by RAFT aqueous polymerization of HEMA in the presence of 0–3 M NaCl.

A pseudo-phase diagram was constructed via TEM analysis by targeting PHEMA DPs of 100–800 in the presence of 0–3 M NaCl (see Figures 5 and S4). In the PISA literature, pseudophase diagrams usually involve systematic variation of the core-forming block DP with either the solids content^28,41,42^ or the stabilizer block DP.^28^ In this study, we chose to vary the PHEMA core-forming block DP with the NaCl concentration (see Figure 5). DLS studies indicated a relatively low scattered light intensity when targeting a relatively short PHEMA DP of 100 in the presence of 0–3 M NaCl, suggesting that only molecularly dissolved diblock copolymer chains are formed under such conditions. This was consistent with TEM analysis since no nanoparticles could be identified during imaging (see Figure 5a). This indicates that RAFT aqueous solution polymerization occurs under such conditions. Similar observations were reported by Ratcliffe and co-workers, who examined the RAFT aqueous polymerization of HEMA at 10% w/w solids using a poly(glycerol monomethacrylate) precursor.^77^ Indeed, molecularly dissolved diblock copolymer chains were obtained even when targeting PHEMA DPs up to 500 in this prior study.

**Scheme 2:**
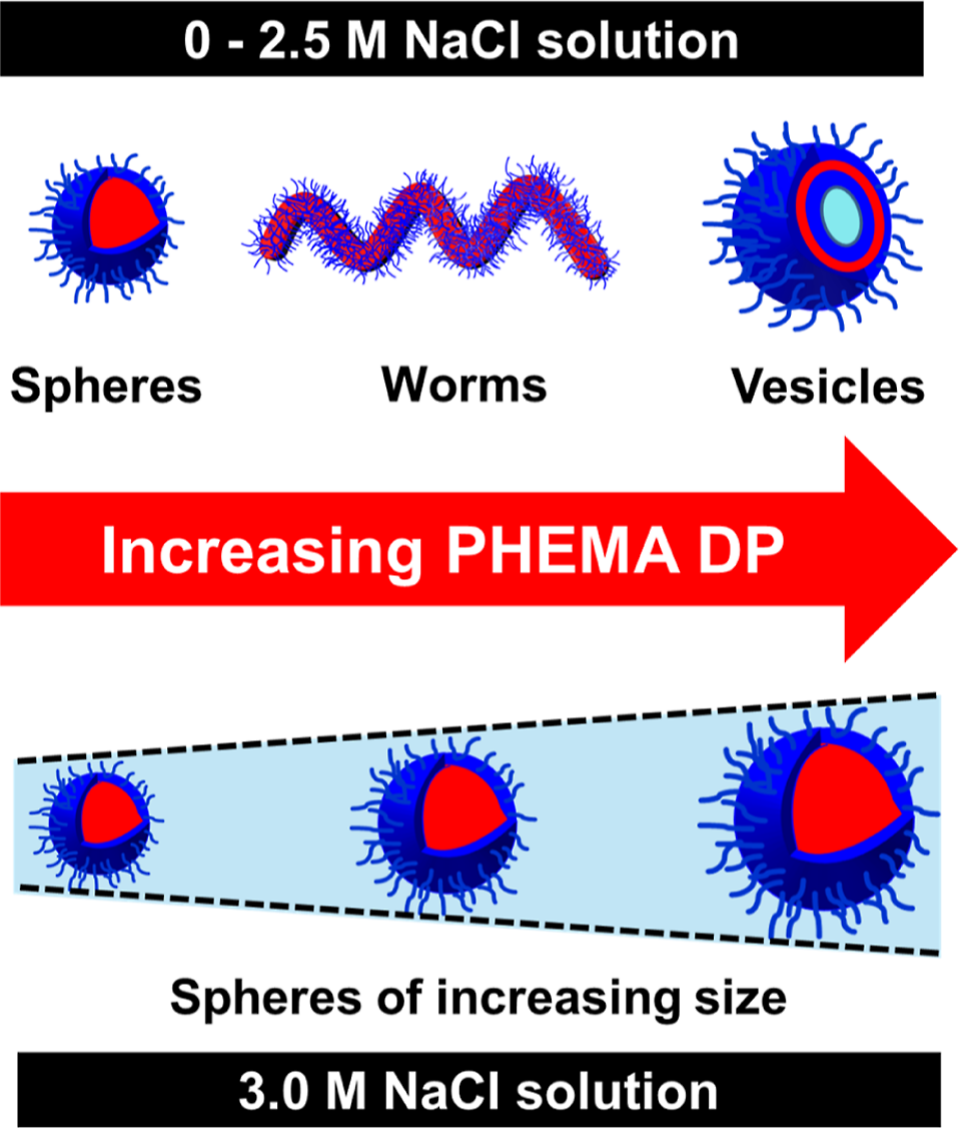
Schematic Representation of the Effect of Added Salt on the RAFT Aqueous Polymerization of HEMA at 70 °C: Systematically Increasing the PHEMA DP Results in a Gradual Evolution in Copolymer Morphology from Spheres to Worms to Vesicles for NaCl Concentrations up to 2.5 M; In Contrast, Only Kinetically Trapped Spheres of Tunable Size Can Be Obtained in the Presence of 3 M NaCl

When targeting higher PHEMA DPs of 200–800, all the three common copolymer morphologies (spheres, worms, and vesicles) could be accessed as pure phases, both in the absence of salt and at all NaCl concentrations up to 2.5 M (see Scheme 2). More specifically, spheres could be accessed when targeting PHEMA DPs ranging from 200 to 400 in the presence of 0–2.5 M NaCl (see Figure 5).

For this copolymer morphology, DLS analysis indicated *z*-average diameters ranging from 25 to 86 nm with relatively low dispersities (PDI = 0.01–0.16). Pure worms could be obtained when targeting PHEMA DPs of either 500 or 600 in the presence of 0–2.5 M NaCl. The apparent *z*-average diameter of such nanoparticles ranged from 118 nm (PDI = 0.09) up to 1728 nm (PDI = 0.60). However, it is emphasized that DLS assumes a spherical morphology, so this technique reports neither the worm contour length nor the worm cross-sectional diameter.

Vesicles were always obtained when targeting PHEMA DPs of either 700 or 800 in the presence of 0–2.5 M NaCl. Hence, the predominant copolymer morphology remains unchanged for salt concentrations up to 2.5 M NaCl, which corresponds to an aqueous dispersion polymerization formulation. However, this PISA formulation resembles an aqueous emulsion polymerization for syntheses conducted in the presence of 3 M NaCl, see Figure 1. Interestingly, only kinetically trapped spheres could be obtained under the latter conditions (see Scheme 2). This morphological limitation is commonly reported for RAFT aqueous emulsion polymerization.^34−37,39−42,55−59,66^ On the other hand, the aqueous solubility of HEMA at 70 °C is still relatively high (∼93 g dm^–3^) in the presence of 3.0 M NaCl. This indicates that only approximately 51–60% of the HEMA becomes water-immiscible when targeting PMPC_26_-PHEMA_200–800_ nanoparticles under such conditions at 20% w/w solids.

**Figure 6:**
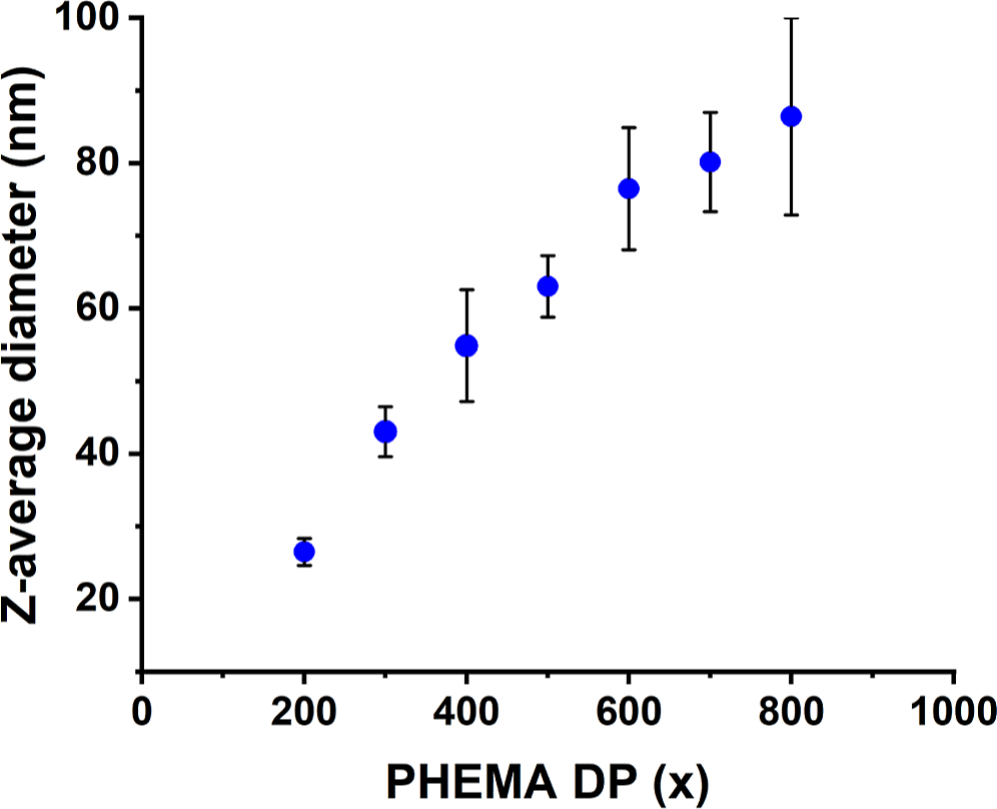
Relationship between *z*-average diameter and core-forming PHEMA DP (*x*) for a series of PMPC_26_-PHEMA_*x*_ (targeting *x* = 200–800) spheres prepared by RAFT aqueous polymerization of HEMA at 70 °C targeting 20% w/w solids in the presence of 3 M NaCl. [N.B. standard deviations are calculated from DLS polydispersities and thus indicate the breadth of each particle size distribution rather than the experimental error].

The relationship between *z*-average diameter and PHEMA DP is shown in Figure 6 for the series of PMPC_26_-PHEMA_*x*_ (*x* = 200–800) spheres produced in the presence of 3 M NaCl. A gradual increase in *z*-average diameter is observed with increasing PHEMA DP. For example, a *z*-average diameter of 26 nm (PDI = 0.07) was determined for PMPC_26_-PHEMA_200_, while PMPC_26_-PHEMA_800_ had a *z*-average diameter of 86 nm (PDI = 0.16). Thus, the nanoparticle diameter can be conveniently controlled simply by adjusting the target PHEMA DP. Similar observations have been reported for various other PISA formulations.^56,57,78^

### Effect of Added NaCl on the Dimensions of PMPC_26_-PHEMA_800_ Vesicles and PMPC_26_-PHEMA_600_ Worms

**Figure 7:**
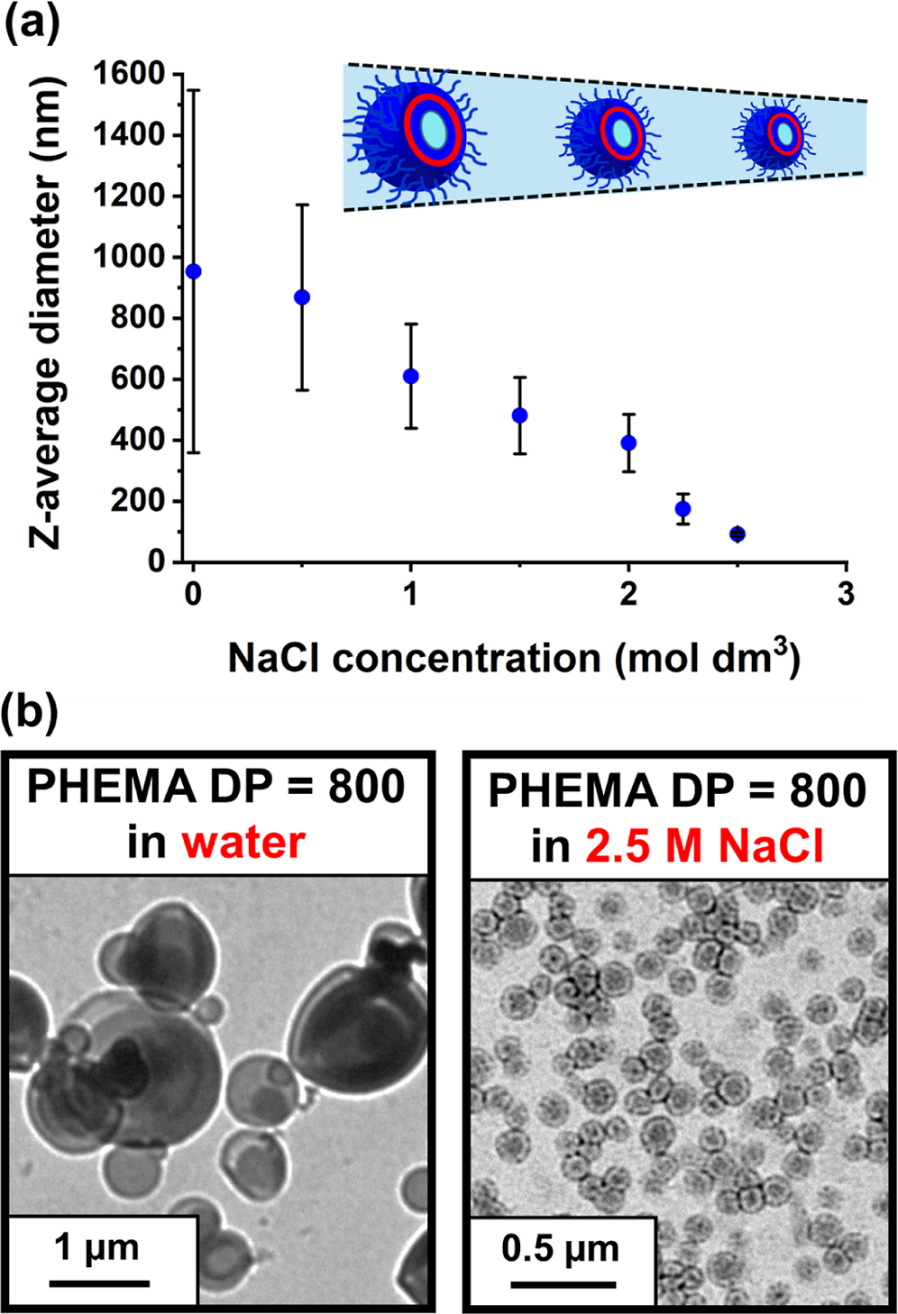
(a) Effect of added salt on the dimensions of PMPC_26_-PHEMA_800_ vesicles as judged by DLS. Standard deviations indicate the breadth of each particle size distribution rather than the experimental error. (b) Corresponding TEM images obtained in the absence of salt and in the presence of 2.5 M NaCl.

As discussed above, the salt concentration has no discernible influence on the copolymer morphology. However, for a series of PMPC_26_-PHEMA_800_ vesicles prepared in the presence of 0–2.5 M NaCl, systematically increasing the salt content led to a gradual reduction in the vesicle diameter. The *z*-average diameter is reduced from 953 nm (PDI = 0.62) in the absence of added salt to 92 nm (PDI = 0.07) in the presence of 2.5 M NaCl (see Figure 7a). Thus, the presence of sufficient salt leads to the formation of relatively small uniform vesicles, whereas vesicles prepared in the absence of salt are relatively large and polydisperse. This remarkable size reduction was confirmed by TEM analysis, see Figure 7b.

This indicates that the nanoparticle dimensions can be conveniently adjusted by simply increasing the NaCl concentration in the aqueous PISA formulation. This is because the addition of salt effectively increases the hydrophobic character of the structure-directing PHEMA chains, which provides a stronger driving force for their self-assembly in aqueous solution.

**Figure 8:**
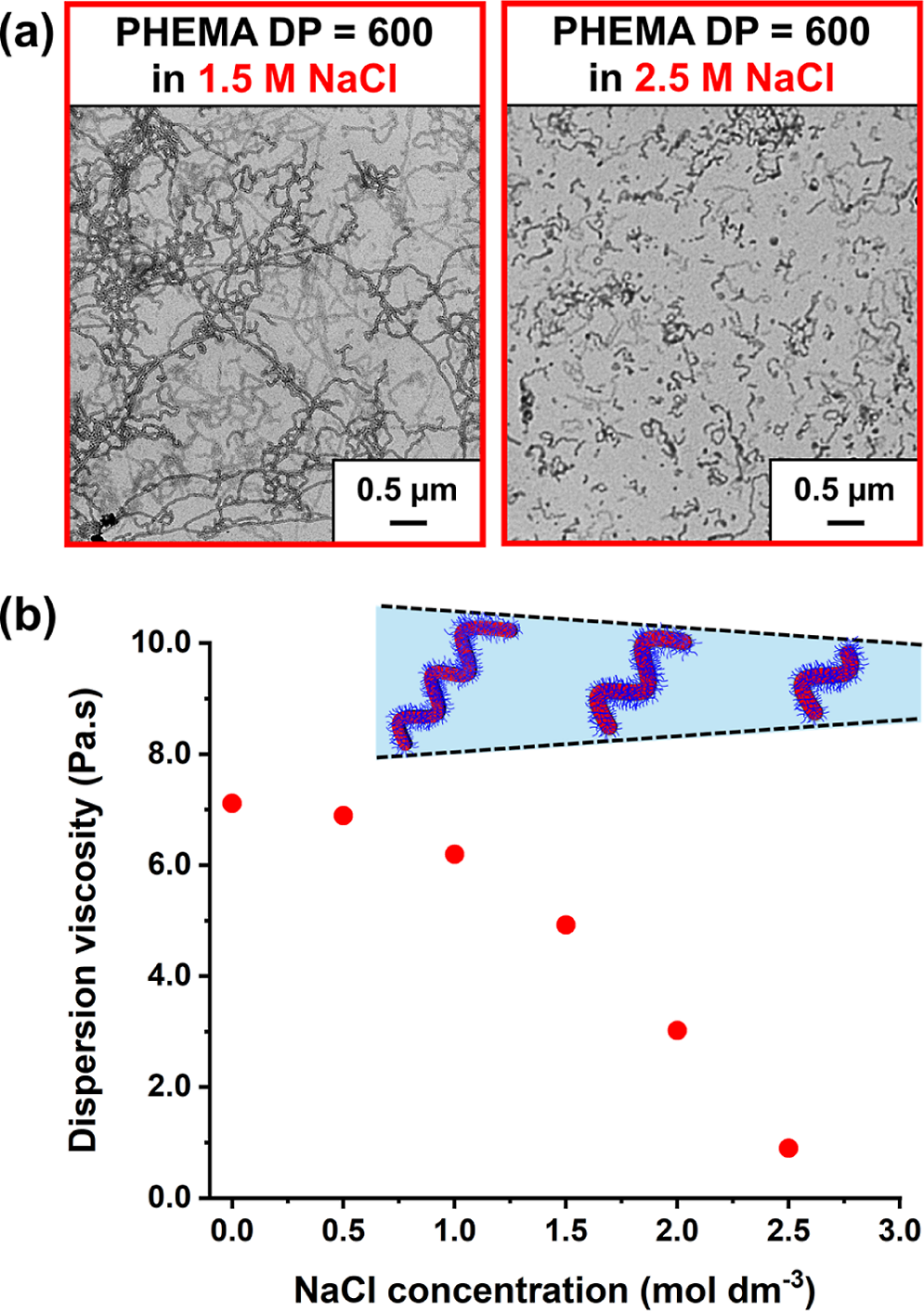
(a) Effect of added NaCl on the dispersion viscosity obtained for PMPC_26_-PHEMA_600_ worms. The dispersion viscosity was determined at a fixed shear rate of 10 s^–1^. (b) Corresponding TEM images obtained for selected salt concentrations (1.5 and 2.5 M NaCl).

TEM analysis of PMPC_26_-PHEMA_600_ worms prepared in the presence of either 1.5 or 2.5 M NaCl suggests a reduction in the mean worm contour length at the higher salt concentration but no significant change in the worm cross-sectional radius (see Figure 8a). This is consistent with the corresponding DLS data (see Figure S5). To confirm this finding, rotational rheometry was used to determine the viscosity of a series of 20% w/w dispersions of PMPC_26_-PHEMA_600_ worms in 0–3 M NaCl at a fixed shear rate of 10 s^–1^ (see Figure 8b). A monotonic reduction in dispersion viscosity was observed from 7.1 Pa s for worms prepared in the absence of salt to 0.9 Pa s for worms prepared in the presence of 2.5 M NaCl. This is consistent with a significant reduction in the mean worm contour length across this series of samples.^79,80^

It is well established that the mean worm cross-sectional diameter is primarily dictated by the core-forming block DP.^30,53,80^ According to Lovett and co-workers, block copolymer worms undergo macroscopic gelation by forming a 3D network of weakly interacting worms. In this case, the critical volume fraction for gelation, Φ_c_, scales as Φ_c_ ∼ *R*/*L*_w_, where *R* is the mean worm cross-sectional radius and *L*_w_ is the mean worm contour length.^81^ If *R* is not significantly affected by added salt, then it follows that the reduction in *L*_w_ indicated by the data shown in Figure 8 should lead to a significant increase in Φ_c_. This is consistent with tube inversion experiments: the relatively short worms produced in the presence of 2.5 M NaCl form a viscous free-flowing fluid, whereas the relatively long worms produced in the presence of 1.5 M NaCl form a free-standing gel (see Figure S6).

Finally, it is perhaps worth mentioning that these PMPC_26_-PHEMA_600_ worms do not exhibit thermoresponsive behavior: no worm-to-sphere transition occurs on cooling to sub-ambient temperature (e.g., 5 °C). This is most likely because the PHEMA DP is too long; similar observations have been reported for PMPC_26_-PHPMA_280_ and PGMA_71_-PHPMA_200_ worms in the PISA literature.^69,82^

## Conclusions

A well-defined PMPC_26_ precursor was chain-extended via RAFT aqueous polymerization of HEMA in the presence of up to 3 M NaCl to yield PMPC_26_-PHEMA_*x*_ (*x* = 200–800) nanoparticles. In situ ^1^H NMR kinetic experiments were conducted to study the synthesis of PMPC_26_-PHEMA_800_ nanoparticles targeting 20% w/w solids at 70 °C. Essentially full HEMA conversion (≥99%) required 6 h in the absence of salt but only 55 min in the presence of 3 M NaCl. This is because the growing PHEMA chains are less soluble under the latter conditions, which leads to the earlier onset of micellar nucleation. GPC analysis indicated a systematic increase in copolymer dispersity when targeting higher PHEMA DPs, particularly at higher salt concentration. This is attributed to dimethacrylate impurities within the HEMA monomer and/or chain transfer to polymer.

A pseudo-phase diagram was constructed for PMPC_26_-PHEMA_*x*_ nanoparticles by systematically varying the PHEMA DP from 100 to 800 as a function of NaCl concentration. TEM was utilized to assess the copolymer morphology. Pure spheres, worms, and vesicles could be obtained when systematically increasing the PHEMA DP at salt concentrations up to 2.5 M NaCl. In contrast, only a series of kinetically trapped spheres could be obtained in the presence of 3 M NaCl. This is because the aqueous solubility of HEMA monomer is significantly lower under the latter conditions, which leads to an aqueous emulsion polymerization formulation. DLS studies confirmed that the *z*-average diameter for this series of spheres increases linearly with the target PHEMA DP.

Finally, TEM and DLS analysis indicate that progressively smaller PMPC_26_-PHEMA_800_ vesicles are produced when increasing the NaCl concentration up to 2.5 M. Similarly, TEM studies suggest that the mean worm contour length of PMPC_26_-PHEMA_600_ worms is significantly reduced at higher salt concentrations, which is consistent with the lower dispersion viscosity. In summary, the addition of NaCl affects the HEMA polymerization kinetics and nanoparticle size but has no discernible influence on the copolymer morphology up to 2.5 M.

## Supporting Information Available

Assigned ^1^H NMR spectrum and aqueous GPC data recorded for the PMPC_26_ precursor; DLS data obtained before and after core-cross-linking for selected PMPC_26_-PHEMA_*x*_ nanoparticles; summary of copolymer characterization data; GPC curves and corresponding *M*_n_ vs. PHEMA DP plot for a series of PMPC_26_-PHEMA_*x*_ copolymers prepared in the presence of 2.5 M NaCl; TEM images for a series of PMPC_26_-PHEMA_*x*_ nanoparticles prepared at various salt concentrations; DLS data for PMPC_26_-PHEMA_600_ worms prepared at various NaCl concentrations; and digital photograph of the physical form of a 20% w/w aqueous dispersion of PMPC_26_-PHEMA_800_ worms prepared in the presence of either 1.5 or 2.5 M NaCl (PDF)
