# Supplementary material for: Effect of Added Salt on the RAFT Polymerization of 2-Hydroxyethyl Methacrylate in Aqueous Media
Source: Macromolecules. 2024 Jul 12;57(14):6816–27. doi: 10.1021/acs.macromol.4c01078 (PMC11271178; doi:10.1021/acs.macromol.4c01078)
Supplement: Supplementary file 1 — ma4c01078_si_001.pdf [file ma4c01078_si_001.pdf]

Supporting Information for:

Effect of added salt on the RAFT polymerization of 2-hydroxyethyl methacrylate in aqueous media

Csilla György[a], Jacob S. Wagstaff[a], Saul J. Hunter[b], Esther U. Etim[a] and Steven P. Armes[a,*]

a. *Dainton Building, Department of Chemistry, Brook Hill, University of Sheffield, Sheffield, South Yorkshire, S3 7HF, UK.*

b. *Joseph Banks Laboratories, School of Chemistry, University of Lincoln, Lincolnshire, LN6 7TS, UK.*

Summary of Contents

Table S1. Summary of DLS data obtained before and after glutaraldehyde crosslinking of the PHEMA core-forming block for a series of $PMPC_{26}$-$PHEMA_x$ nanoparticles.

Figure S1. Assigned ^{1}H NMR spectrum recorded in CD_3OD for the $PMPC_{26}$ precursor prepared *via* RAFT solution polymerization in ethanol using the CPDB RAFT agent.

Figure S2. Aqueous GPC curve recorded for the $PMPC_{26}$ precursor prepared via RAFT solution polymerization in ethanol at 70 °C when targeting 40% w/w solids.

Table S2. Summary of the GPC, DLS, and TEM data obtained for a series of $PMPC_{26}$-$PHEMA_{100-800}$ nano-objects prepared at 20% w/w solids.

Table S3. Summary of the GPC, DLS, and TEM data obtained for a series of $PMPC_{26}$-$PHEMA_{100-800}$ nano-objects prepared at 20% w/w solids in 0.5 M NaCl solution.

Table S4. Summary of the GPC, DLS, and TEM data obtained for a series of $PMPC_{26}$-$PHEMA_{100-800}$ nano-objects prepared at 20% w/w solids in 1 M NaCl solution.

Table S5. Summary of the GPC, DLS, and TEM data obtained for a series of $PMPC_{26}$-$PHEMA_{100-800}$ nano-objects prepared at 20% w/w solids in 1.5 M NaCl solution.

Table S6. Summary of the GPC, DLS, and TEM data obtained for a series of $PMPC_{26}$-$PHEMA_{100-800}$ nano-objects prepared at 20% w/w solids in 2 M NaCl solution.

Table S7. Summary of the GPC, DLS, and TEM data obtained for a series of $PMPC_{26}$-$PHEMA_{100-800}$ nano-objects prepared at 20% w/w solids in 2.5 M NaCl solution.

Table S8. Summary of the GPC, DLS, and TEM data obtained for a series of $PMPC_{26}$-$PHEMA_{100-800}$ nano-objects prepared at 20% w/w solids in 3 M NaCl solution.

Figure S3. (a) GPC curves (*vs.* a series of near-monodisperse poly(methyl methacrylate) calibration standards using a refractive index detector) obtained for the $PMPC_{26}$ precursor and a series of $PMPC_{26}$-$PHEMA_{100-800}$ diblock copolymers. **(b)** Linear relationship between M_n (blue circles) and PHEMA DP for the same $PMPC_{26}$-$PHEMA_{100-800}$ series (plus M_w/M_n data).

Figure S4. TEM images obtained for $PMPC_{26}$-$PHEMA_{300}$, $PMPC_{26}$-$PHEMA_{600}$ and $PMPC_{26}$-$PHEMA_{800}$ nanoparticles prepared in the presence of 1.0, 1.5 M, 2.0, 2.5 and 3.0 M NaCl.

Figure S5. Effect of added salt on the 'sphere-equivalent' z-average diameter of $PMPC_{26}$-$PHEMA_{600}$ worms as judged by DLS.

Figure S6. Digital photograph recorded for 20% w/w aqueous dispersions of $PMPC_{26}$-$PHEMA_{800}$ worms prepared in the presence of either 1.5 M NaCl or 2.5 M NaCl.

Table S1. Summary of DLS data obtained before and after glutaraldehyde crosslinking of the PHEMA core-forming block for a series of $PMPC_{26}$-$PHEMA_x$ nanoparticles prepared at 20% w/w solids in 2.5 M NaCl solution. TEM morphologies are also included for reference.

Target composition	DLS Before Crosslinking		DLS After Crosslinking		TEM Morphology
	D_h (nm)	PDI	D_h (nm)	PDI	
$PMPC_{26}$-$HEMA_{200}$	53	0.21	58	0.24	Spheres
$PMPC_{26}$-$HEMA_{300}$	47	0.25	52	0.23	Spheres/worms
$PMPC_{26}$-$HEMA_{600}$	170	0.24	118	0.23	Worms
$PMPC_{26}$-$HEMA_{700}$	96	0.22	82	0.05	Vesicles
$PMPC_{26}$-$HEMA_{800}$	92	0.07	85	0.05	Vesicles

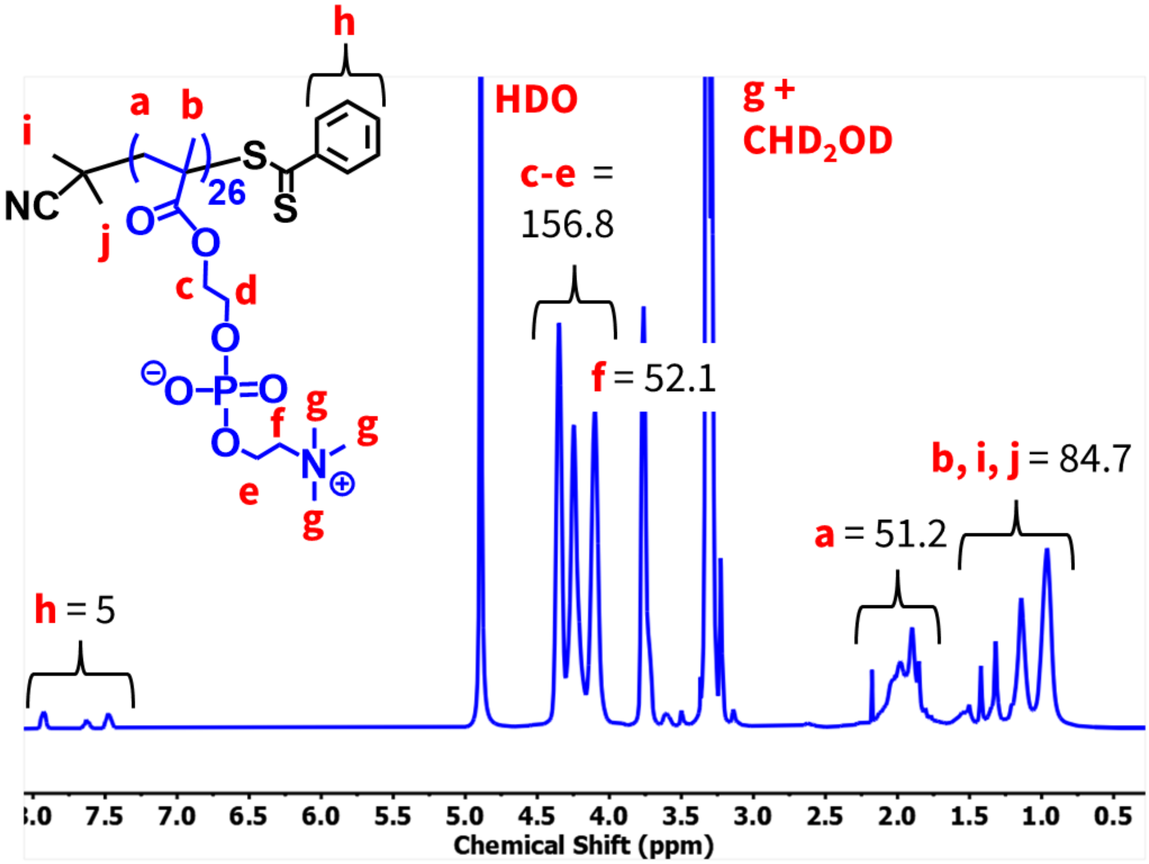

Figure S1. Assigned ^{1}H NMR spectrum (with integrated signals) recorded in CD_3OD for the $PMPC_{26}$ precursor prepared *via* RAFT solution polymerization in ethanol using the CPDB RAFT agent.

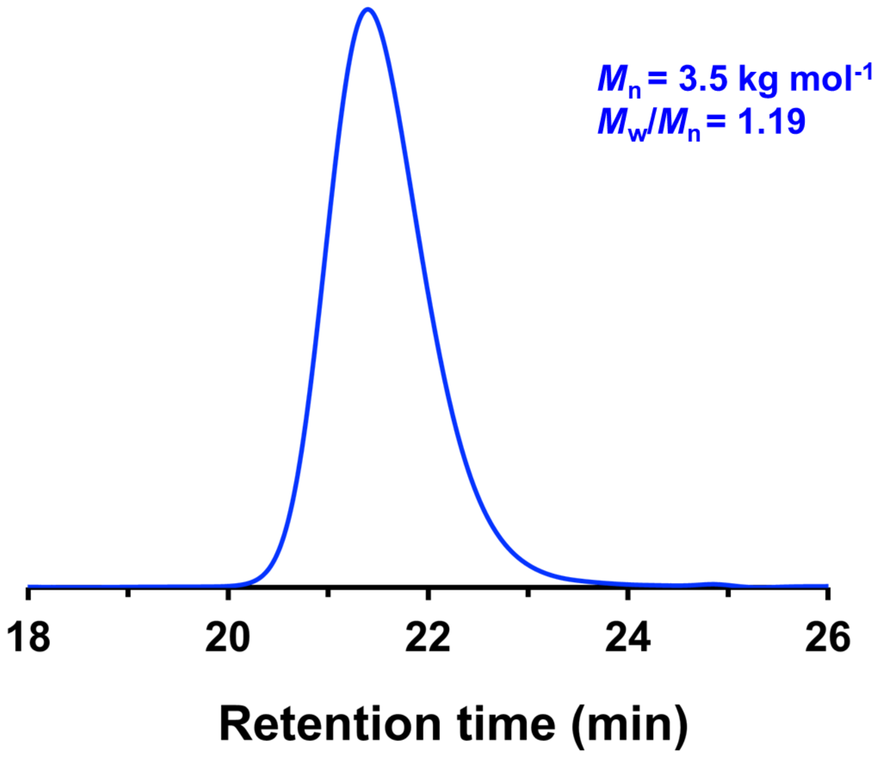

Figure S2. Aqueous GPC curve recorded for the $PMPC_{26}$ precursor (prepared via RAFT solution polymerization in ethanol at 70 °C when targeting 40% w/w solids).

Table S2. Summary of the GPC, DLS, and TEM data obtained for a series of $PMPC_{26}$-$PHEMA_{100-800}$ diblock copolymer nano-objects prepared at 20% w/w solids in water. The $PMPC_{26}$ precursor is also included as a reference.

Target composition	Chloroform/methanol GPC		DLS		TEM Morphology
	M_n (kg mol^{-1})	M_w/M_n	D_h (nm)	PDI	
$PMPC_{26}$	3.6	1.32	N/A	N/A	N/A
$PMPC_{26}$-$HEMA_{100}$	12.3	1.35	108	0.78	Chains
$PMPC_{26}$-$HEMA_{200}$	22.7	1.38	22	0.16	Chains
$PMPC_{26}$-$HEMA_{300}$	37.0	1.56	31	0.04	Spheres
$PMPC_{26}$-$HEMA_{400}$	49.8	1.43	36	0.06	Spheres
$PMPC_{26}$-$HEMA_{500}$	64.4	1.50	98	0.24	Spheres/worms
$PMPC_{26}$-$HEMA_{600}$	68.4	1.75	1728	0.60	Worms
$PMPC_{26}$-$HEMA_{800}$	94.2	1.72	953	0.62	Vesicles

Table S3. Summary of the GPC, DLS, and TEM data obtained for a series of $PMPC_{26}$-$PHEMA_{100-800}$ diblock copolymer nano-objects prepared at 20% w/w solids in 0.5 M NaCl solution.

Target composition	Chloroform/methanol GPC		DLS		TEM Morphology
	M_n (kg mol^{-1})	M_w/M_n	D_h (nm)	PDI	
$PMPC_{26}$-$HEMA_{100}$	13.5	1.23	275	0.46	Chains
$PMPC_{26}$-$HEMA_{200}$	23.3	1.29	25	0.14	Spheres
$PMPC_{26}$-$HEMA_{300}$	33.4	1.36	38	0.02	Spheres
$PMPC_{26}$-$HEMA_{400}$	51.1	1.28	44	0.01	Spheres
$PMPC_{26}$-$HEMA_{500}$	55.6	1.44	131	0.28	Spheres/worms
$PMPC_{26}$-$HEMA_{600}$	60.8	1.59	981	0.98	Worms
$PMPC_{26}$-$HEMA_{800}$	N/A	N/A	869	0.35	Vesicles

Table S4. Summary of the GPC, DLS, and TEM data obtained for a series of $PMPC_{26}$-$PHEMA_{100-800}$ diblock copolymer nano-objects prepared at 20% w/w solids in 1 M NaCl solution.

Target composition	Chloroform/methanol GPC		DLS		TEM Morphology
	M_n (kg mol^{-1})	M_w/M_n	D_h (nm)	PDI	
$PMPC_{26}$-$HEMA_{100}$	14.5	1.25	117	0.32	Chains
$PMPC_{26}$-$HEMA_{200}$	25.0	1.30	27	0.08	Spheres
$PMPC_{26}$-$HEMA_{300}$	42.4	1.23	40	0.01	Spheres
$PMPC_{26}$-$HEMA_{400}$	49.7	1.39	127	0.22	Spheres/worms
$PMPC_{26}$-$HEMA_{500}$	66.9	1.43	343	0.24	Spheres/worms
$PMPC_{26}$-$HEMA_{600}$	83.8	1.53	583	0.60	Worms
$PMPC_{26}$-$HEMA_{800}$	93.5	2.24	610	0.28	Vesicles

Table S5. Summary of the GPC, DLS, and TEM data obtained for a series of $PMPC_{26}$-$PHEMA_{100-800}$ diblock copolymer nano-objects prepared at 20% w/w solids in 1.5 M NaCl solution.

Target composition	Chloroform/methanol GPC		DLS		TEM Morphology
	M_n (kg mol^{-1})	M_w/M_n	D_h (nm)	PDI	
$PMPC_{26}$-$HEMA_{100}$	13.4	1.39	405	0.59	Chains
$PMPC_{26}$-$HEMA_{200}$	24.8	1.32	31	0.03	Spheres
$PMPC_{26}$-$HEMA_{300}$	37.3	1.37	44	0.06	Spheres
$PMPC_{26}$-$HEMA_{400}$	60.9	1.34	169	0.26	Spheres/worms
$PMPC_{26}$-$HEMA_{500}$	67.9	1.56	440	0.27	Worms
$PMPC_{26}$-$HEMA_{600}$	79.5	1.84	540	0.34	Worms
$PMPC_{26}$-$HEMA_{800}$	149.8	2.69	481	0.26	Vesicles

Table S6. Summary of the GPC, DLS, and TEM data obtained for a series of $PMPC_{26}$-$PHEMA_{100\text{-}800}$ diblock copolymer nano-objects prepared at 20% w/w solids in 2 M NaCl solution.

Target composition	Chloroform/methanol GPC		DLS		TEM Morphology
	M_n (kg mol^{-1})	M_w/M_n	D_h (nm)	PDI	
$PMPC_{26}$-$HEMA_{100}$	12.7	1.36	171	0.56	Chains
$PMPC_{26}$-$HEMA_{200}$	22.2	1.29	33	0.16	Spheres
$PMPC_{26}$-$HEMA_{300}$	33.4	1.29	40	0.04	Spheres
$PMPC_{26}$-$HEMA_{400}$	45.1	1.44	69	0.14	Spheres/worms
$PMPC_{26}$-$HEMA_{500}$	62.6	1.71	237	0.29	Worms
$PMPC_{26}$-$HEMA_{600}$	78.4	1.50	509	0.20	Worms
$PMPC_{26}$-$HEMA_{800}$	88.8	2.05	391	0.24	Vesicles

Table S7. Summary of the GPC, DLS, and TEM data obtained for a series of $PMPC_{26}$-$PHEMA_{100\text{-}800}$ diblock copolymer nano-objects prepared at 20% w/w solids in 2.5 M NaCl solution.

Target composition	Chloroform/methanol GPC		DLS		TEM Morphology
	M_n (kg mol^{-1})	M_w/M_n	D_h (nm)	PDI	
$PMPC_{26}$-$HEMA_{100}$	9.6	1.28	18710	0.59	Chains
$PMPC_{26}$-$HEMA_{200}$	22.7	1.32	53	0.21	Spheres
$PMPC_{26}$-$HEMA_{300}$	33.2	1.40	47	0.25	Spheres/worms
$PMPC_{26}$-$HEMA_{400}$	53.0	1.31	61	0.11	Spheres/worms
$PMPC_{26}$-$HEMA_{500}$	57.8	1.77	118	0.09	Worms
$PMPC_{26}$-$HEMA_{600}$	60.7	1.81	170	0.24	Worms
$PMPC_{26}$-$HEMA_{700}$	95.8	2.39	96	0.22	Vesicles
$PMPC_{26}$-$HEMA_{800}$	101.6	2.73	92	0.07	Vesicles

Table S8. Summary of the GPC, DLS, and TEM data obtained for a series of $PMPC_{26}$-$PHEMA_{100\text{-}800}$ diblock copolymer nano-objects prepared at 20% w/w solids in 3 M NaCl solution.

Target composition	Chloroform/methanol GPC		DLS		TEM Morphology
	M_n (kg mol^{-1})	M_w/M_n	D_h (nm)	PDI	
$PMPC_{26}$-$HEMA_{100}$	6.7	1.31	331	0.50	Spheres
$PMPC_{26}$-$HEMA_{200}$	14.3	1.57	26	0.07	Spheres
$PMPC_{26}$-$HEMA_{300}$	25.8	1.38	43	0.08	Spheres
$PMPC_{26}$-$HEMA_{400}$	46.1	1.71	55	0.14	Spheres
$PMPC_{26}$-$HEMA_{500}$	53.3	1.69	63	0.11	Spheres
$PMPC_{26}$-$HEMA_{600}$	71.9	2.97	76	0.11	Spheres
$PMPC_{26}$-$HEMA_{700}$	88.3	1.68	80	0.09	Spheres
$PMPC_{26}$-$HEMA_{800}$	103.4	3.07	86	0.16	Spheres

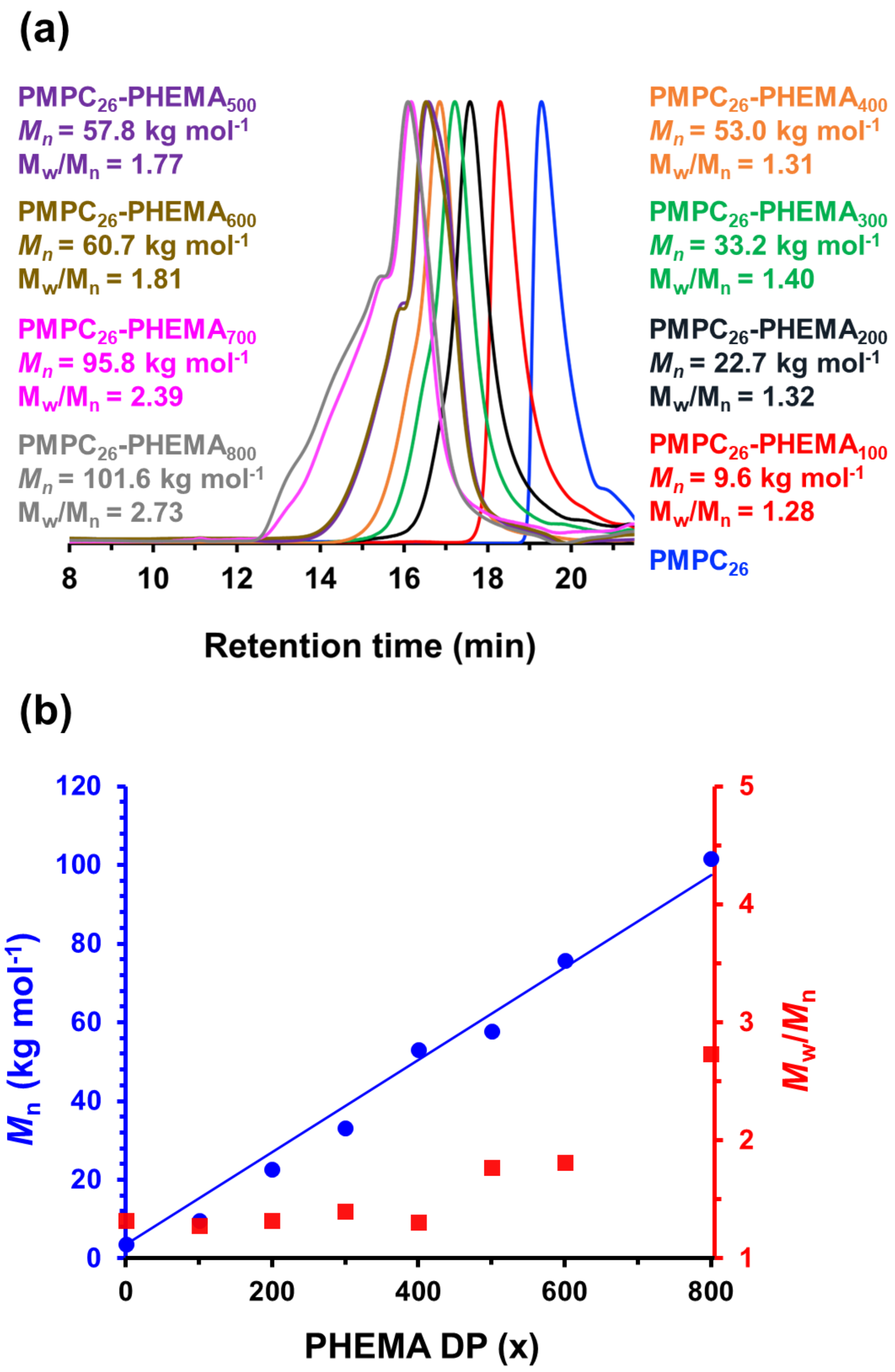

Figure S3. (a) Gel permeation chromatograms (*vs.* a series of near-monodisperse poly(methyl methacrylate) calibration standards using a refractive index detector) obtained for the $PMPC_{26}$ precursor (prepared in ethanol at 40% w/w solids at 70 °C) and a series of $PMPC_{26}$-$PHEMA_{100\text{-}800}$ diblock copolymers prepared by RAFT aqueous dispersion polymerization of HEMA at 70 °C targeting 20% w/w solids in the presence of 2.5 M NaCl. **(b)** Linear relationship between M_n (blue circles) and PHEMA DP for the same $PMPC_{26}$-$PHEMA_{100\text{-}800}$ series. The corresponding M_w/M_n (red squares) data are also shown.

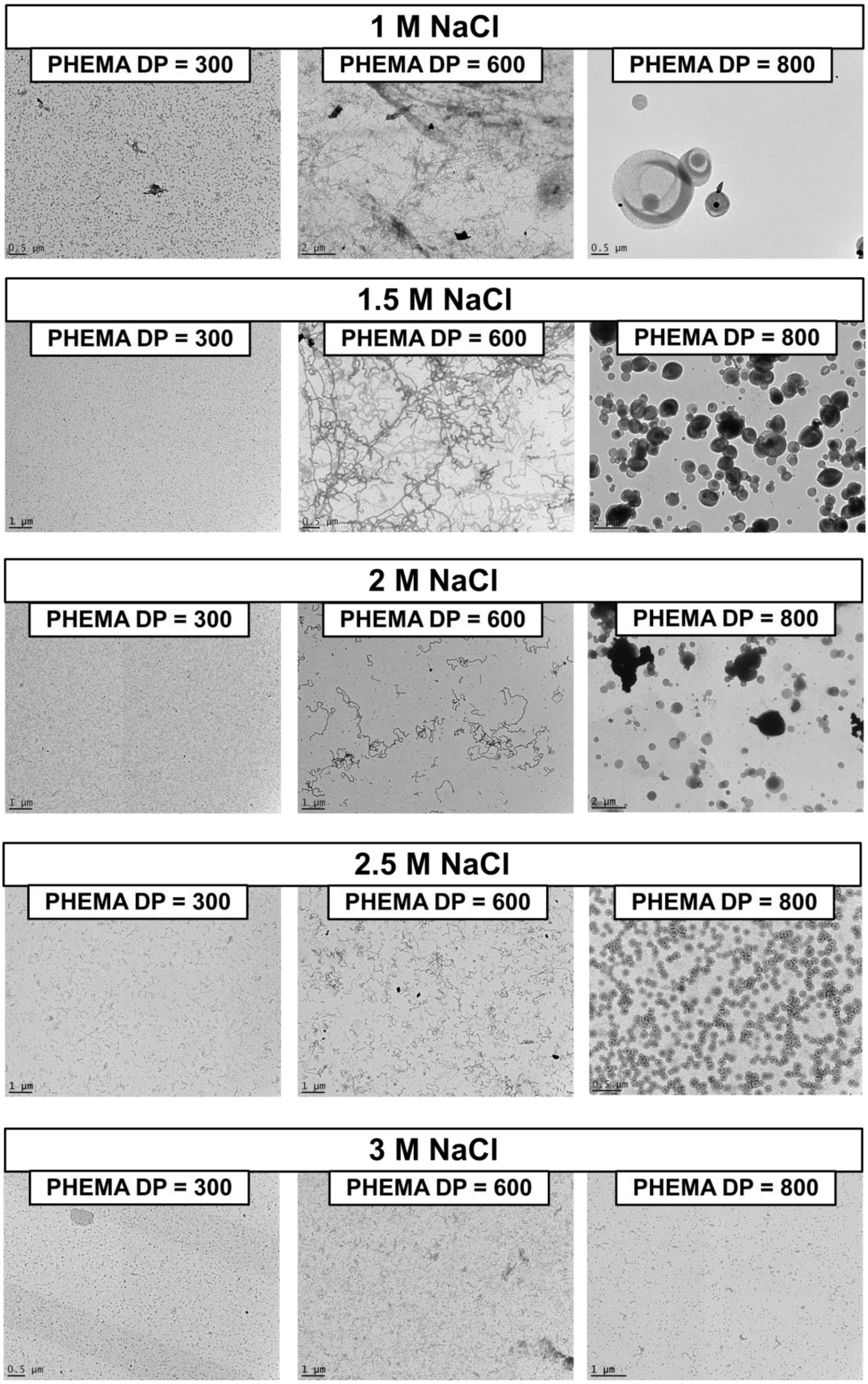

Figure S4. Representative TEM images obtained for $PMPC_{26}$-$PHEMA_{300}$, $PMPC_{26}$-$PHEMA_{600}$ and $PMPC_{26}$-$PHEMA_{800}$ nanoparticles prepared in 1.0 M, 1.5 M, 2.0 M, 2.5 M and 3.0 M NaCl solution when targeting 20% w/w solids at 70 °C.

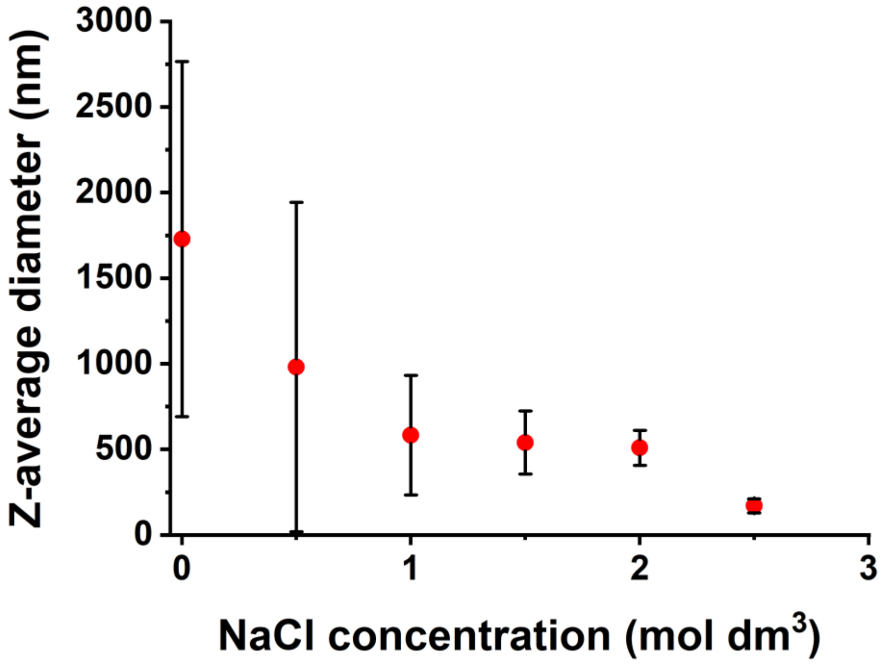

Figure S5. Effect of added salt on the 'sphere-equivalent' z-average diameter of $PMPC_{26}$-$PHEMA_{600}$ worms as judged by DLS. Standard deviations indicate the breadth of each particle size distribution, rather than the experimental error. The z-average diameter corresponds to neither the worm contour length nor the worm width. Nevertheless, the reduction in this parameter can be used to monitor the relative change in worm dimensions as a function of added salt.

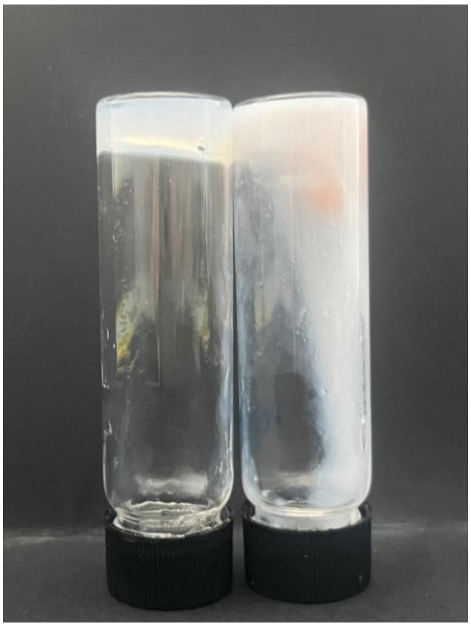

Figure S6. Digital photograph recorded for 20% w/w aqueous dispersions of $PMPC_{26}$-$PHEMA_{800}$ worms prepared in the presence of either 1.5 M NaCl (left, free-standing gel) or 2.5 M NaCl (right, viscous fluid).
